# Mechanical Properties of Fully Recyclable 3D-Printable Materials Used for Application in Patient-Specific Devices in Radiotherapy

**DOI:** 10.3390/polym17141946

**Published:** 2025-07-16

**Authors:** Antonio Jreije, Paulius Griškevičius, Neringa Keršienė, Jurgita Laurikaitienė, Rūta Nedzinskienė, Diana Adlienė

**Affiliations:** 1 Department of Physics, Kaunas University of Technology, Studentu Str. 50, 51368 Kaunas, Lithuania; jurgita.laurikaitiene@ktu.lt; 2 Department of Mechanical Engineering, Kaunas University of Technology, Studentų Str. 56, 51424 Kaunas, Lithuania; paulius.griskevicius@ktu.lt (P.G.); neringa.kersiene@ktu.lt (N.K.); 3 Bioeconomy Research Institute, Vytautas Magnus University, Studentu Str. 11, Akademija, 53361 Kaunas, Lithuania; ruta.nedzinskiene@ktu.lt

**Keywords:** 3D-printable composites, recycling, mechanical properties, patient specific equipment, radiotherapy

## Abstract

The exponential growth of plastic production in the healthcare sector and the limited capacity of conventional recycling systems have created a global environmental challenge. Latest 3D printing technologies have the potential to solve this problem by enabling on-demand, localized manufacturing. This study aimed to investigate the mechanical properties of 3D-printed ABS composites with Bi_2_O_3_ fillers after multiple recycling and irradiation cycles to assess their suitability for creating robust, reusable supporting devices for radiotherapy. Filaments of PLA, ABS, and ABS composites enriched with 5 wt% and 10 wt% Bi_2_O_3_ were extruded, repeatedly recycled through shredding and re-extrusion up to ten times and irradiated to 70 Gy using a 6 MeV photon beam to simulate clinical radiotherapy conditions. In contrast to PLA, ABS demonstrated better recyclability; however, after ten recycling cycles, its tensile strength declined from 25.1 MPa to 20.9 MPa, and its Young’s modulus decreased from 2503.5 MPa to 1410.4 MPa. Incorporation of 5 wt% Bi_2_O_3_ into ABS significantly improved recyclability and mechanical retention. After ten recycling rounds, an ABS composite containing 5 wt% Bi_2_O_3_ retained tensile strength of 22.2 MPa, modulus of 1553.9 MPa, and strain at break of 14.4%. In contrast, the composite enforced with 10 wt% Bi_2_O_3_ showed slightly lower performance, likely due to filler agglomeration. Under irradiation, the ABS–5 wt% Bi_2_O_3_ composite exhibited minimal additional degradation, maintaining mechanical integrity superior to other materials. These results indicate that ABS–5 wt% Bi_2_O_3_ is a promising, recyclable material for durable, patient-specific devices in radiotherapy, supporting sustainability in medical manufacturing.

## 1. Introduction

Plastic waste has become a pressing global environmental concern, with the healthcare sector recognized as a significant contributor. In 2020, global plastic production exceeded 380 million tons, yet only about 16% of plastic waste was recycled [1]. Healthcare systems rely heavily on single-use plastic items, including syringes, catheters, tubing, and sterile packaging, to maintain hygiene and safety standards. This dependence results in substantial plastic consumption, with healthcare facilities worldwide estimated to use approximately 15 million tons of plastic annually [2]. According to the World Health Organization, up to 55% of hospital general waste consists of plastics, but only around 20% of this waste is effectively recycled [3]. This reliance on disposables not only generates immense waste but also carries a carbon footprint due to energy-intensive manufacturing and incineration processes [4]. These environmental challenges are driving a search for more sustainable practices in medicine, including better waste management and material reuse.

Additive manufacturing, commonly referred to as 3D printing, has emerged as a transformative technology in modern healthcare, offering both clinical and sustainability advantages [5]. Unlike traditional subtractive manufacturing, 3D printing builds objects layer by layer, potentially minimizing excess material and waste during production [6,7,8,9]. Over the past decade, the medical sector has rapidly adopted 3D printing due to its ability to produce on demand, patient-specific devices with high precision and short turnaround times [10]. Hospitals now routinely employ in-house 3D printing to fabricate custom anatomical models for surgical planning, tailored surgical guides, prosthetic components, and even implants, all designed to match individual patient anatomies [11].

This personalized approach has been shown to improve treatment accuracy and patient outcomes, particularly in complex clinical scenarios. The integration of point-of-care 3D printing is expanding quickly in leading hospitals, thus further embedding the technology into routine clinical workflows [11]. In cardiovascular medicine, for instance, more than 200 studies have reported the use of 3D-printed, patient-specific phantoms for procedural planning, simulation, and training [12]. While these advances illustrate the growing role of 3D printing in delivering personalized care, they also introduce new challenges related to the lifecycle and sustainability of the materials employed.

The field of radiation oncology has been among the earliest medical specialties to integrate 3D printing into clinical workflows, particularly for the development of patient-specific solutions [13,14]. The use of 3D printing enables the production of highly individualized phantoms derived from patient imaging data (e.g., CT/MRI scans), thus improving the accuracy of dosimetric validation [15]. Kamomae et al., 2017, successfully fabricated a patient-specific head and neck phantoms incorporating anatomical features such as bone structures, air cavities, and sinus structures using patient CT datasets [16]. Similarly, Huynh et al. (2022) created a custom larynx phantom based on a volunteer’s MRI images to simulate the airway and tumor motion during MR-guided radiotherapy [17].

Beyond anatomical phantoms, additive manufacturing has been used to create personalized radiotherapy accessories, including bolus devices conforming to irregular patient surfaces, immobilization masks, brachytherapy applicator molds, and compensators for beam modulation [18]. These innovations improve treatment accuracy, patient comfort, and reproducibility by ensuring a closer anatomical fit compared to conventional alternatives. Multiple international cancer centers have successfully implemented 3D-printed boluses at scale. Basaula et al. (2024) reported the fabrication of over 2000 custom polylactic acid (PLA) boluses between 2018 and 2023, while demonstrating consistent Hounsfield units (~80 HU) and establishing an efficient quality control program to ensure dosimetric accuracy [19]. Clinical studies further confirm that 3D-printed boluses made from Acrylonitrile Butadiene Styrene (ABS) improve dose coverage of the skin and chest wall in breast cancer radiotherapy while maintaining good conformity and tolerable skin toxicity [20]. These clinical adoption underscores that 3D-printed boluses are not merely theoretical innovations but are already contributing to improved clinical outcomes in routine patient care.

However, most of these 3D-printed items are single-use by design [18,19]. Because they are customized for individual patients or procedures and may be subject to contamination, they are typically discarded after a single application for safety and practical reasons. As the adoption of patient-specific devices continues to rise, this practice is contributing to a new stream of plastic waste, raising concerns about sustainability and the urgent need for viable recycling or reuse strategies in clinical settings.

Despite increasing clinical adoption of 3D-printed accessories in radiotherapy, there remains a significant gap in systematic research addressing the sustainability and end-of-life management of commonly used printing materials, particularly under conditions that simulate real-world clinical reuse. While existing studies have primarily focused on the feasibility and dosimetric performance of 3D-printed phantoms and boluses, to the best of our knowledge [10,11,12,13,14,15,16,17,18,19,20,21], no previous work has examined whether these materials can be effectively recycled or how their mechanical and functional properties evolve after repeated processing and exposure to ionizing radiation. In our previous work, we demonstrated that acrylonitrile butadiene styrene (ABS) composites enhanced with high atomic number (Z) fillers such as bismuth oxide (Bi_2_O_3_) exhibit favorable mechanical performance and radiation stability under therapeutic photon doses, supporting their suitability for radiotherapy applications [22]. However, questions regarding the recyclability, structural integrity, and long-term usability of such materials remain largely unanswered. Addressing these issues is essential, as it places 3D printing at the intersection of medical device innovation and circular economy principles—where material reuse, waste reduction, and environmental sustainability are becoming increasingly important in modern healthcare.

In order to address this concern, the present work examines the mechanical properties of ABS composites with bismuth oxide (Bi_2_O_3_) fillers after multiple recycling and irradiation cycles, comparing their performance with standard FDM materials—ABS and PLA. By evaluating the durability and resiliency of recycled composites, this study assesses their suitability for creating robust, reusable supporting devices for radiotherapy and demonstrates the compatibility of 3D printing materials’ recycling with the general goals of the circular economy.

## 2. Materials and Methods

### 2.1. Production of 3D Printing Filaments

ABS and PLA thermoplastics were chosen as matrix materials to produce 3D-printable filament composites. The pellet form of the materials was purchased from 3Devo Filament Maker, Utrecht, The Netherlands. Bi_2_O_3_ powder, <200 nm (99.999% trace metals basis), chosen as a composite filer, was purchased from Sigma Aldrich (Taufkirchen, Germany).

Based on the findings of our previous study [22], ABS composites were formulated by incorporating Bi_2_O_3_ at concentrations of 5 wt% and 10 wt%. Bismuth oxide was selected due to its high atomic number and proven efficacy in photon attenuation, making it suitable for radiological applications. In this study, these filler loadings were found to achieve an optimal compromise between enhanced radiation shielding, mechanical performance, and filament processability, factors critical for the practical implementation of 3D-printed components in radiotherapy settings [22].

PLA and ABS pellets were extruded into 1.75 ± 0.1 mm diameter filaments using a single screw extruder (Precision 350, 3Devo Filament Maker, The Netherlands). The extrusion temperature for ABS was adjusted as follows: preheating (240 °C), melting (230 °C), shear (220 °C), and extrusion (215 °C). The selected extrusion temperatures for PLA were: 170 °C, 185 °C, 190 °C, and 170 °C. Filament spooling was performed after the extruded filament became stable.

The same extrusion procedure was applied twice when producing ABS composites, containing 5 wt% or 10 wt% of Bi_2_O_3_ additives. In the first step, 3D filaments were extruded from the dry mixture of the components (ABS pellets and Bi_2_O_3_ powders). To secure better homogeneity of the final composites, in the second step, produced filaments were shredded and ground into small flakes (regrinds) using a shredder 3DEVO SHR3D IT (Utrecht, The Netherlands) and dried for a couple of hours at 50 °C. In the third step, 1.75 ± 0.1 mm diameter 3D filament composites were extruded again, using the same parameters as in the previous extrusion step.

### 2.2. Recycling and Reprocessing of 3D Printing Materials

In the first recycling step, irradiated and non-irradiated pieces of plastic were crushed in the shredder SHR3D IT into small flakes suitable for extrusion. Prior to the extrusion process, the granule regrinds were dried in a Creality filament dry box (Creality 3D Technology Co., Ltd., Shenzhen, China) (heating temperature of 50 °C) for 2 h while periodically stirring the material to ensure even moisture release. This was performed to prevent hydrolysis caused by the prolonged exposure of polymers to elevated temperatures in the filament extruder such as 3Devo, as it typically takes the material approximately 10 to 15 min to travel across the screw from the hopper to the nozzle. Finally, extrusion was performed using 3Devo Filament Maker, The Netherlands.

In order to simplify and speed up the recycling process, the extruded filaments were recycled directly by shredding and extruding them again. This process of filament shredding and extruding was repeated 10 times to imitate a real case of 5 recycling cycles and 20 times as equivalent to the real case of 10 recycling cycles. This experimental methodology is highly accurate since the extrusion temperature of ABS using 3Devo is as high as 240 °C while 3D printing temperature of ABS is 230–270 °C. Therefore, ABS is exposed to a similar processing temperature during filament fabrication and printing. The same was true for PLA, where the processing temperature was in the range of 180–220 °C.

### 2.3. 3D Printing of Samples

Differently shaped samples were printed from the produced filaments. For the investigation of radiation density and fillers distribution within polymer matrix, 3D-printed samples of the size 50 × 50 × 5 mm^3^ were used. For mechanical evaluation of the virgin and recycled 3D printing thermoplastics and composite materials, tensile specimens (dog bone) and bending specimens (rectangular cross-section) were printed according to the ISO 527-2:2025 [23] standard (type 1A) and ISO 178:2019 [24], respectively. Printing of samples was performed using a commercial 3D printer Zortrax M300 (Olsztyn, Poland). The printing parameters were set as follows: 220–270 °C printing temperature, 100% solid infill, 0.29 mm layer thickness, and 80 °C build plate temperature.

### 2.4. Irradiation of Samples

Irradiation of samples was performed in a linear accelerator (Clinac DMX, Varian, Palo Alto, CA, USA). Samples were irradiated up to the total dose of 70 Gy while using 6 MeV photons, which corresponds to the possible total irradiation dose delivered to the tumor during intensity-modulated radiation therapy (IMRT) treatment of a broad variety of cancers.

### 2.5. Evaluation of Radiation Hardness and Homogeneity of Samples

To assess internal structural homogeneity and potential filler agglomeration, computed tomography (CT) scans were performed using a GE Revolution HD scanner (GE Healthcare, Chicago, IL, USA) with the following scanning parameters: tube voltage of 120 kVp, current of 200 mA, and a slice thickness of 1.25 mm. CT numbers were evaluated using ImageJ software version 1.54 (NIH, Bethesda, MD, USA) by selecting a 20 mm^2^ region of interest (ROI) over five consecutive slices to calculate average Hounsfield Units (HUs) and standard deviations.

Mass attenuation coefficients for the composite materials were estimated using the XCOM database provided by the National Institute of Standards and Technology (NIST) [25]. Simulations were carried out to assess the attenuation behavior across a range of X-ray photon energies, with a focus on energy ranges relevant to diagnostic imaging and radiotherapy. Comparative analyses were performed against standard materials such as soft tissue and polycaprolactone. For soft tissue, the elemental composition was based on the ICRU-44 reference model, which consists primarily of oxygen (76.2%), carbon (11.1%), hydrogen (10.2%), and nitrogen (2.6%), with trace amounts of other elements [26]. This standard tissue surrogate is widely used in medical physics to approximate the radiological properties of average adult soft tissue [26]. A summary of the elemental compositions and densities for all materials used in the simulations is provided in Table 1.

### 2.6. Evaluation of Mechanical Properties

**Figure 1:**
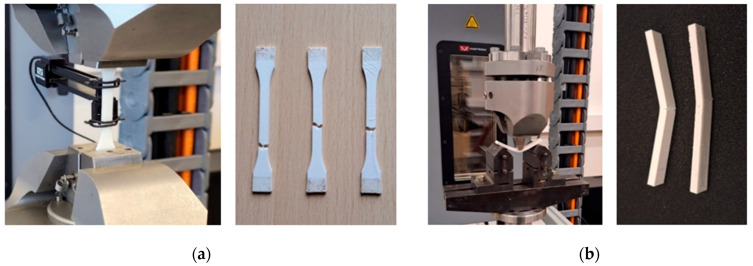
Tensile test (**a**) and flexural test (**b**) of ABS composite samples.

Evaluation of the mechanical properties of the newly proposed and 3D-printed samples as well as the impact of high energy X-ray irradiation on the mechanical properties of these samples was the main issue in analyzing the composite’s ability to serve as a material for 3D-printed devices in radiation therapy. The tensile and flexural strength, strain at fracture and other mechanical characteristics of the 3D-printed samples were investigated using an ElectroPuls^®^ E10000 Linear-Torsion machine (Instron, MA, USA) according to ISO 527-1:2019 [27] and ISO 178:2019 [24], respectively (Figure 1).

Statistical analysis was performed to evaluate the reproducibility of mechanical test results. For each material and condition, three specimens were tested. The mean and standard deviation (SD) were calculated for all measured parameters, including tensile strength, Young’s modulus, and strain at break. If the standard deviation exceeded 15% of the mean, additional specimens were tested to ensure consistency. Data analysis and visualization were conducted using Microsoft Excel.

## 3. Results and Discussion

### 3.1. Characterization of 3D-Printed and Recycled Materials

**Figure 2:**
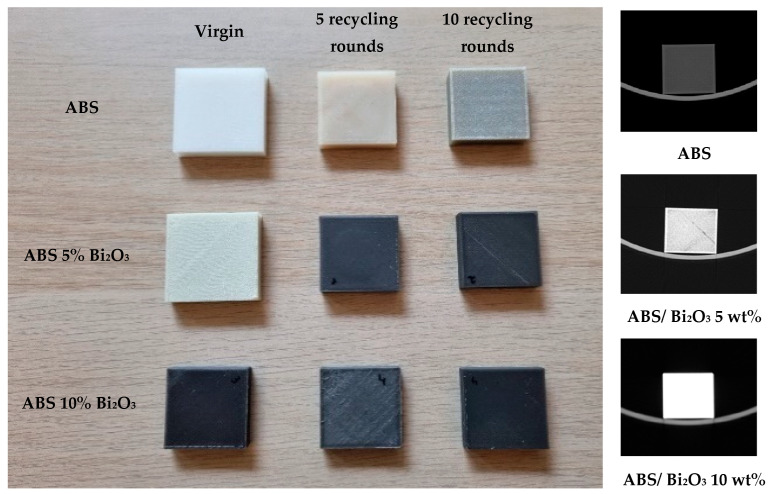
Visual appearance of 3D-printed samples after various recycling rounds (**Left**) and CT scans of virgin samples (**Right**).

The following in-house produced materials were recycled: PLA, ABS, ABS/ Bi_2_O_3_ 5 wt%, and ABS/ Bi_2_O_3_ 10 wt%. PLA was successfully recycled both once and twice. Various failed attempts were made to recycle PLA for a third round, but each attempt yielded a brittle filament of inconsistent diameter with a high number of visible air bubbles. We also extruded 3D filaments from the mixture of 2/3 virgin PLA and 1/3 recycled PLA. On the other hand, ABS and ABS-based composites were successfully recycled five times and 10 times. Examples of virgin and recycled samples are shown in Figure 2 together with CT images of these samples.

The visual inspection of the samples printed from recycled ABS and ABS/Bi_2_O_3_ composites revealed a gradual color change and darkening of the samples over multiple recycling rounds. This observation can be attributed to increased thermal stress effects in the thermoplastic. The molecular structure of recycled polymer undergoes alterations during mechanical recycling and high-temperature stages in filament production and deposition [28]. These changes, such as reductions in molecular weight, can impact viscosity and the final mechanical properties. For example, Zhao et al. detected the thermal degradation of PLA through repeated recycling cycles, with molecular chain scission identified as the primary degradation mechanism via Fourier-transform infrared spectroscopy (FTIR). This degradation led to alterations in rheology, crystallinity, and morphology of the printed components [29]. Similarly, Rahimi et al., 2014 indicated that ABS undergoes degradation with each recycling cycle in injection molding applications [30]. Bai et al., 2007, identified the chemical degradation using FTIR, through an increase in peak intensity between 1680 and 1750 cm^−1^, indicating the formation of carbonyl groups [31]. Additionally, differential scanning calorimetry analysis of injection molded ABS revealed a significant decrease in average molecular weight and an increase in the polydispersity index (PDI) following recycling [32].

### 3.2. Modelling of X-Ray Atenuation Properties of Samples

X-ray attenuation properties of polymer composites enriched with metals/metal oxides can be particularly advantageous in medical imaging or radiation therapy applications, where precise control over radiation absorption is crucial. However, it is requested that the radiation attenuation properties of materials used to print out phantoms and boluses or patient-specific supporting devices in radiotherapy be similar to those of biological tissues and not interfere with the delivery of the prescribed treatment dose to the tumor.

**Figure 3:**
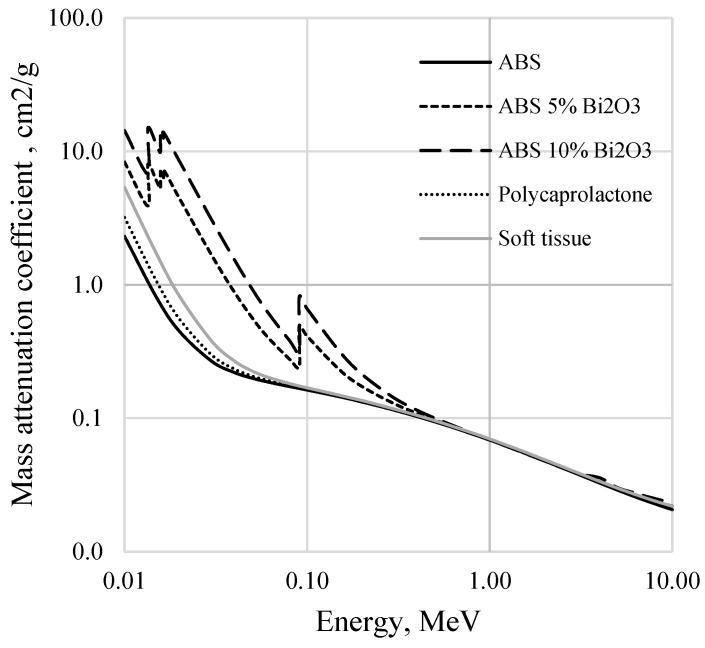
NIST XCOM simulated mass attenuation coefficients of the newly developed 3D printing filaments. The attenuation of human soft tissue and polycaprolactone (commercial immobilization mask’s material) are indicated for comparison.

As can be seen from Figure 3, ABS itself is almost tissue equivalent, having a steadily decreasing mass attenuation coefficient with the increased X-ray energy. ABS/Bi_2_O_3_ composites demonstrated significantly high absorption, especially near the Bi K-edge, which occurs at 90.5 keV, due to the photoelectric interaction of photons with material, characterizing a Bi_2_O_3_-enriched composite as an effective shielding material against low-energy photons. Beyond the K-edge, mass attenuation coefficients of these composites were decreasing with the increasing energy similar to those of pure ABS or biological tissue, indicating reduced shielding effectiveness because other energy loss processes different from photoelectric absorption became more important at higher energies.

### 3.3. Mechanical Performance After Recycling

**Figure 4:**
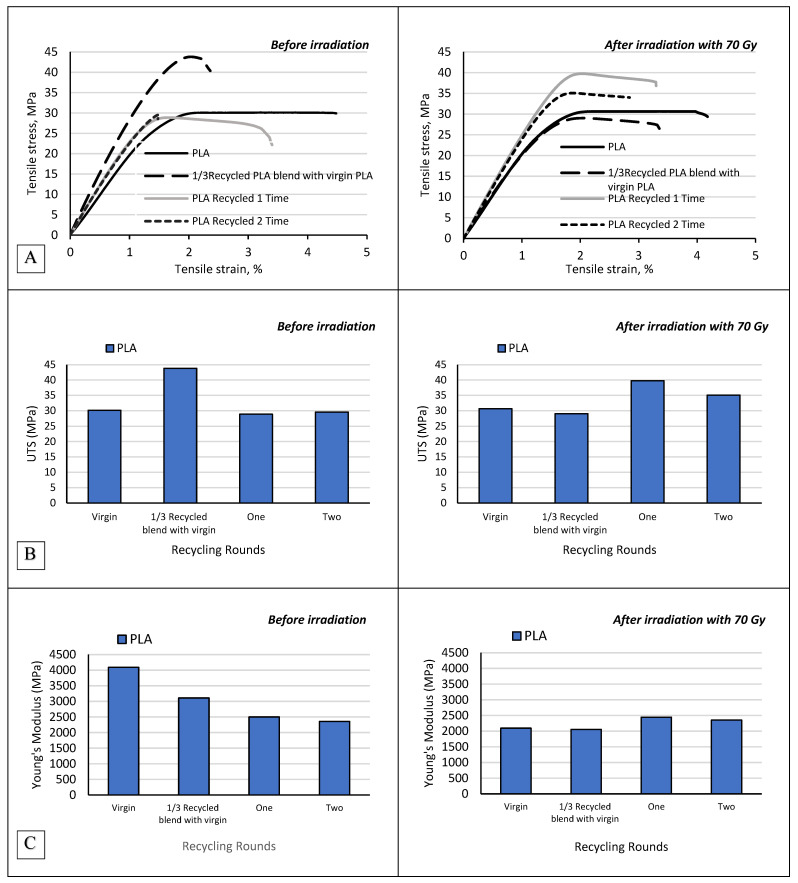
Tensile properties of non-irradiated and irradiated PLA specimens before and after recycling: (**A**)—stress and strain graph; (**B**)—Ultimate Tensile Stress (UTS); (**C**)—Young’s Modulus.

Mechanical properties of the newly fabricated 3D-printable materials were investigated to assess the influence of recycling and the impact of high dose (70 Gy) irradiation on composites performance as patient specific supporting materials in radiotherapy. Tensile and flexural tests were performed on both irradiated and non-irradiated virgin and recycled ABS and PLA filaments and ABS/Bi_2_O_3_ composites. The impact of recycling and irradiation on the mechanical properties of the investigated PLA specimens is presented in Figure 4. Across the materials tested, significant differences emerged in tensile strength, Young’s Modulus, and strain at break, which became particularly pronounced with successive recycling cycles. For PLA, initial values for tensile stress at maximum load (30.13 ± 2.75 MPa) and Young’s modulus (4091.65 ± 245.1 MPa) decreased notably after two recycling cycles, reaching 29.56 ± 2.41 MPa and 2356.8 ± 201.5 MPa, respectively. This decline suggests PLA’s limited capacity for recycling, with reductions in tensile strength and modulus reflecting polymer chain scission and degradation under repeated thermal processing. This is consistent with the literature indicating that PLA experiences molecular breakdown and embrittlement more rapidly than ABS, especially under thermal stress from extrusion processes [33,34].

On the other hand, mixing virgin PLA with recycled PLA improved its mechanical properties. The UTS of 1/3 recycled PLA blend with virgin PLA increased to 43.8 ± 3.87 MPa as compared to 30.13 ± 2.75 MPa for virgin PLA, while the strain at maximum load increased from 1.6% for virgin PLA to 2% for 1/3 recycled PLA blend with virgin PLA. On the other hand, the YM was lower for 1/3 recycled PLA blend with virgin PLA (3.1 ± 0.21 GPa vs. 4.1 GPa ± 0.24 for virgin PLA). These findings can be explained by the partial incorporation of recycled PLA, which introduces a moderate level of chain scission and molecular weight reduction. In pure recycled PLA, chain scission typically leads to brittleness and reduced strength; however, when blended with virgin PLA, the recycled component can act as a toughening phase. This toughening is due to the interactions between short chain recycled polymers and the longer chains in virgin PLA, which enhance the composite’s ability to distribute stress and prevent premature fracture. The increase in UTS suggests that this balance between the recycled and virgin components improves load-bearing capacity and resistance to breakage under tension.

When subjected to 70 Gy of irradiation, both virgin and recycled PLA materials showed a significant reduction in Young’s modulus, indicative of radiation-induced polymer chain scission, which may reduce stiffness. Young’s modulus of virgin PLA decreased drastically from 4091.65 ± 245.1 MPa to 2095.42 ± 198.3 MPa post-irradiation. However, the effect of irradiation on tensile strength varied depending on the recycling stage. Virgin PLA’s tensile strength remained relatively stable after irradiation (increasing slightly from 30.13 ± 2.75 MPa to 30.67 ± 3.56 MPa), while recycled PLA showed a more complex behavior. Notably, PLA recycled once exhibited an increase in tensile strength from 28.88 ± 2.63 MPa to 39.76 ± 4.12 MPa after irradiation. While such an increase might suggest the occurrence of radiation-induced crosslinking, this is unlikely given the low irradiation dose used in this study (70 Gy), which is significantly below the thresholds typically required to induce measurable crosslinking in PLA—often reported in the range of 30–50 kGy [35]. Instead, the improved tensile strength is more likely attributed to annealing-like effects such as partial molecular orientation, relaxation of internal stresses, or enhanced interlayer bonding, phenomena previously observed in thermally or mildly irradiated PLA [36,37].

**Figure 5:**
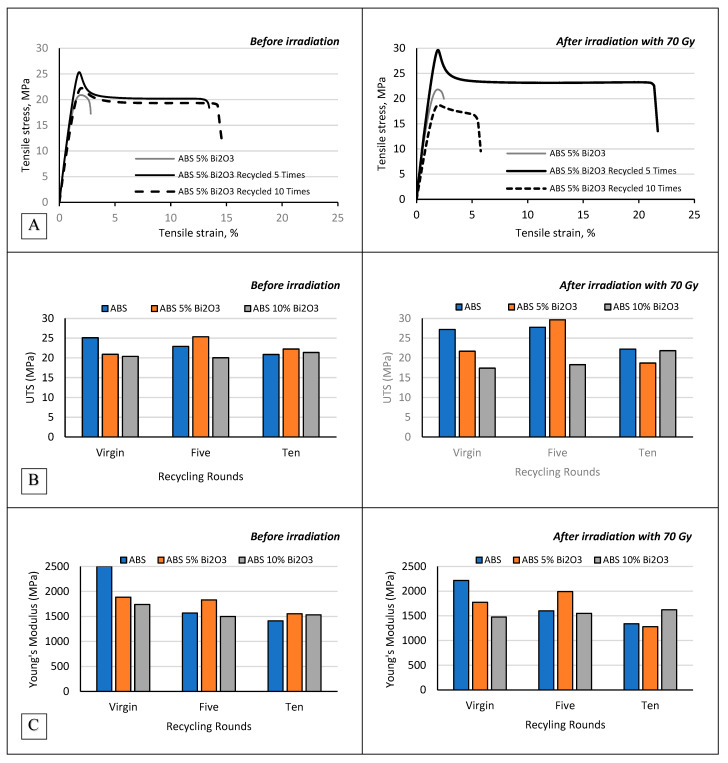
Tensile properties of non-irradiated and irradiated ABS samples, including composites with 5% and 10% Bi_2_O_3_, evaluated before recycling and after 5 and 10 recycling cycles. (**A**) Representative stress–strain curves for each material condition. (**B**) Ultimate Tensile Strength (UTS) values. (**C**) Young’s Modulus values. Each data point represents the average of three tested specimens per condition. Specimen groups are differentiated by Bi_2_O_3_ filler content (0%, 5%, or 10%), number of recycling cycles (0, 5, or 10), and irradiation status (irradiated vs. non-irradiated).

Tensile properties of non-irradiated and irradiated ABS and ABS composites enriched with Bi_2_O_3_ before and after recycling are shown in the graphs provided in Figure 5.

ABS, while showing improved recyclability compared to PLA, still exhibited declines in mechanical properties with each recycling round. The tensile stress at maximum load for virgin ABS, initially 25.1 ± 1.98 MPa, dropped to 20.86 ± 1.16 MPa after ten cycles, while Young’s modulus decreased from 2503.5 ± 127.2 MPa to 1410.4 ± 115.9 MPa. The reduction in tensile properties in pure ABS suggests that repeated processing leads to thermal degradation, likely causing chain scission, which, in turn, decreases stiffness and strength. However, the relatively moderate decline compared to PLA demonstrates ABS’s greater suitability for recycling, as it retains more of its mechanical properties after multiple cycles. Nonetheless, this level of degradation still limits its viability for high-durability applications.

Similar results were observed by Cress et al. who investigated the effect of three recycling cycles on the properties of 3D-printable ABS [38]. In this study, recycling of used samples was performed by granulation of experimental samples in a micro-cut paper shredder. By the third round of recycling, Tensile Stress (TS) decreased by 11%, strain at tensile strength (εTS) by 13%, and fracture stress (σ_f_) by 8%. Young’s moduli for both virgin and recycled batches were statistically similar, having approximately 1.6 GPa value. Recycling also impacted strain at fracture (ε_f_), with an approximate 8% reduction observed with each recycling cycle, resulting in a 25% decrease from virgin ABS to ABS recycled by three times [38]. These reductions were also reflected in a roughly 37% decrease in toughness by the third round of recycling. According to this study, the primary factor responsible for decreased tensile properties of recycled ABS was the heightened porosity post-recycling. The authors reported an increase in porosity from 11 vol% to 16–18 vol% post-recycling [29].

The introduction of Bi_2_O_3_ fillers in ABS markedly improved the retention of mechanical properties after multiple recycling cycles. The ABS 5% Bi_2_O_3_ composite displayed tensile stress at a maximum load of 22.24 ± 1.67 MPa and Young’s Modulus of 1553.91 ± 108.6 MPa after ten recycling cycles. These values were significantly higher than those of pure ABS under the same conditions. Additionally, the strain at break for ABS 5% Bi_2_O_3_ remained high (14.437%), indicating sustained ductility. These findings suggest that the presence of Bi_2_O_3_ fillers in ABS helps to mitigate some of the thermal degradation effects, possibly by reinforcing the polymer matrix and slowing down the rate of molecular breakdown. The observed increase in ductility in ABS 5% Bi_2_O_3_ composites compared to virgin and recycled ABS is particularly beneficial in fabrication of patient-specific radiotherapy devices, which require materials that can withstand handling and strain without fracturing.

In contrast, the ABS 10% Bi_2_O_3_ composite, while also showing enhanced mechanical properties relative to pure ABS, demonstrated a slight decline in tensile performance after multiple recycling cycles compared to the 5% Bi_2_O_3_ blend. After ten cycles, the tensile stress at maximum load and Young’s modulus were slightly lower (21.37 ± 2.12 MPa and 1533.01 ± 142.7 MPa, respectively) than those of the ABS 5% Bi_2_O_3_ composite. This trend may be attributed to filler agglomeration at higher concentrations, which can introduce microstructural inconsistencies, reducing the composite’s overall stability and performance. Thus, while the ABS 10% Bi_2_O_3_ composite performs better than pure ABS, the ABS 5% Bi_2_O_3_ composite presents a more balanced reinforcement without the drawbacks of excessive filler content.

The effects of irradiation on ABS and ABS composites were found to be minimal, even after ten recycling cycles. Tensile strength and Young’s modulus values remained largely unchanged following exposure to 70 Gy, indicating that high-energy irradiation did not significantly degrade the mechanical properties of either pure ABS or Bi_2_O_3_ reinforced composites. This trend was consistent across all tested materials, suggesting that the addition of Bi_2_O_3_ did not lead to increased susceptibility to radiation damage.

**Figure 6:**
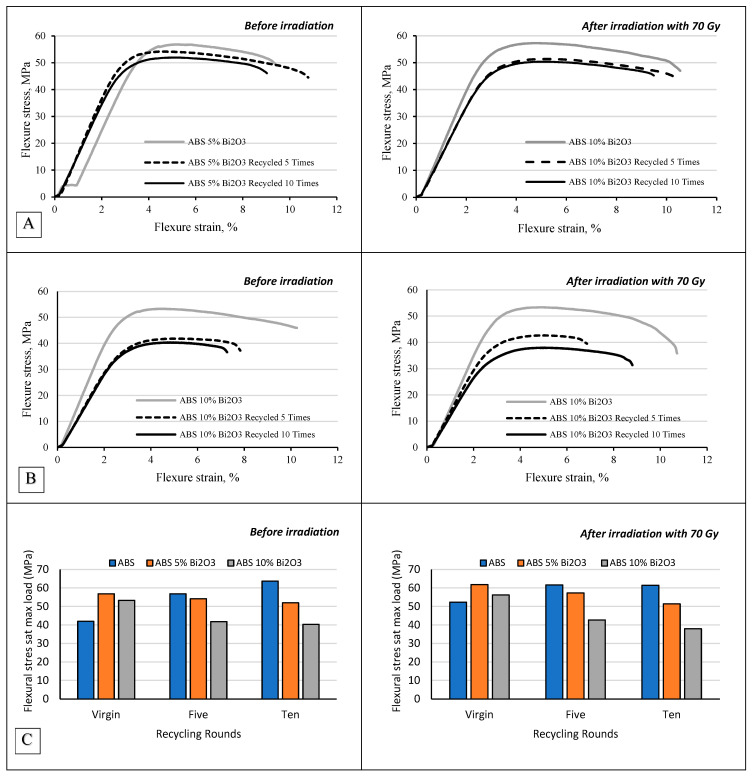
Flexural properties of non-irradiated and irradiated ABS samples, including composites with 5% and 10% Bi_2_O_3_, evaluated before recycling and after 5 and 10 recycling cycles. (**A**,**B**) Representative stress–strain curves under three-point bending for each material condition. (**C**) Flexural stress at maximum load. Each data point represents the average of three specimens per condition. Specimen groups are defined by Bi_2_O_3_ content (0%, 5%, or 10%), number of recycling cycles (0, 5, or 10), and irradiation status (irradiated vs. non-irradiated).

The flexural performance of the materials further highlighted the benefits of Bi_2_O_3_-filled ABS composites (Figure 6). The flexural stress at maximum load for pure ABS increased from 41.95 ± 4.54 MPa in its virgin state to 63.68 ± 4.96 MPa after 10 recycling cycles. This considerable decline in flexural strength underscores the material’s decreasing capacity to bear bending stress, which can be problematic in applications requiring dimensional stability under load. However, it was found that the ABS 5% Bi_2_O_3_ composite retained a higher level of flexural stress at maximum load (51.96 ± 5.16 MPa) after 10 rounds of recycling, showing significantly better retention of flexural integrity than pure ABS. Moreover, the flexural strain at break for the ABS 5% Bi_2_O_3_ composite remained relatively high across recycling rounds, indicating that the material continued to exhibit favorable bending performance and resilience even after extended recycling. This property is especially advantageous for radiotherapy applications (e.g., fixation masks), where materials are subjected to repeated positioning and stress, as it suggests a lower risk of fracture under repeated use. In contrast, the ABS 10% Bi_2_O_3_ composite exhibited a slight decline in flexural performance relative to the ABS 5% Bi_2_O_3_ composite after multiple cycles, likely due to increased filler interactions and potential stress concentration points formed by filler agglomeration.

## 4. Conclusions

In conclusion, this study demonstrates that the mechanical properties of PLA, ABS, and home-made ABS composites with Bi_2_O_3_ fillers are significantly influenced by recycling and irradiation, with each material showing distinct responses to these factors. PLA, although commonly used in 3D printing, exhibited the least resilience to recycling, with rapid declines in tensile strength, ductility, and stiffness after only two cycles. Pure ABS, though more recyclable, also experienced considerable reductions in mechanical performance after 10 recycling rounds making it less ideal for applications demanding high durability.

The addition of Bi_2_O_3_ fillers to ABS notably enhanced its mechanical stability under both recycling and irradiation conditions. The ABS 5% Bi_2_O_3_ composite, in particular, maintained favorable tensile and flexural properties across multiple recycling rounds, with high strain at break values and limited declines in strength and stiffness even after prolonged exposure to thermal degradation and radiation. In contrast, the ABS 10% Bi_2_O_3_ composite showed slightly reduced performance due to potential filler agglomeration, indicating that lower filler concentrations are more effective in preserving material uniformity and strength.

For applications in radiotherapy, particularly in the production of phantoms and immobilization devices, the ABS 5% Bi_2_O_3_ composite stands out as an optimal material. This composite’s favorable balance of tensile strength, flexibility, and radiation resistance makes it suitable for creating durable, patient-specific devices that can be reused across multiple treatment sessions, supporting both patient safety and environmental sustainability.

Overall, the findings underscore the potential of tailored ABS composites in advancing sustainable, high-performance materials for medical applications within a circular economy framework. Future research should focus on long-term performance under clinical use conditions, and expansion to other polymer systems or additive formulations tailored for radiological performance and recyclability. Additionally, integration into clinical workflows should be explored by validating the practical use of these recycled materials in real-world radiotherapy settings through pilot studies involving 3D-printed phantoms and immobilization tools used during treatment planning and delivery.

## Figures and Tables

**Table 1:** Elemental composition and density parameters used in XCOM simulations for radiation attenuation analysis.

Material	Elemental Composition (% by Weight)	Density (g/cm^3^)
Human Soft Tissue (ICRU 44) [26]	H (10.5%), C (11.1%), N (2.6%), O (76.3%)	1.04
Polycaprolactone	H (9%), C (63%), O (28%)	1.15
ABS	H (8%), C (86%), N (2%), O (4%)	0.99
ABS 10% Bi_2_O_3_	H (8%), C (77%), N (2%), O (4%), Bi (10%)	1.26

## Data Availability

The original contributions presented in the study are included in the article. Further inquiries can be directed to the corresponding author.

## Abbreviations

The following abbreviations are used in this manuscript:

ABS	Acrylonitrile butadiene styrene
Bi_2_O_3_	Bismuth oxide
CT	Computed tomography
FDM	Fused deposition modeling
FTIR	Fourier-transform infrared spectroscopy
Gy	Gray
HU	Hounsfield unit
MRI	Magnetic resonance imaging
PDI	polydispersity index
PLA	Polylactic acid
3D	Three-dimensional
